# Problems and Strategies of Allocating Public Service Resources in Rural Areas in the Context of County Urbanization

**DOI:** 10.3390/ijerph192114596

**Published:** 2022-11-07

**Authors:** Muzhe Pan, Yaofu Huang, Yawen Qin, Xun Li, Wei Lang

**Affiliations:** 1 School of Geography and Planning, Sun Yat-sen University, Guangzhou 510275, China; 2 China Regional Coordinated Development and Rural Construction Institute, Sun Yat-sen University, Guangzhou 510275, China

**Keywords:** county urbanization, equalization of public services, trend of population mobility, planning strategies, urban-rural integration

## Abstract

Imbalances in allocating public service resources are a universal problem worldwide, especially in urban and rural areas. As a developing country with a significant imbalance between urban and rural areas, China is representative of the unbalanced allocation of public service resources. Presently, China has entered the county urbanization stage. Therefore, this study can provide a new way to realize the equalization of urban and rural public services with the county as the basic unit. Taking counties as the primary study area, this paper analyzes the new trends of population mobility in China’s counties. It combines large-scale questionnaires and field surveys to investigate the new demand of rural residents for public services and the shortcomings of public service resource allocation. First, the county seat attracts a concentration of the county’s rural residents and returning population, whose high expectations for the county seat’s education and medical services have not yet been met. Second, the township assumes the vital function of elementary school education and medical services in rural areas, and the rural children have a great demand for elementary school education services in the township. However, there are still apparent shortcomings in support of teaching facilities and the quality of education services. Third, the problem of aging and hollowing out in rural areas is serious, and the education, medical and elderly service needs of left-behind children and the elderly are difficult to be guaranteed. Finally, this paper proposes targeted planning strategies and policy recommendations for allocating county public service resources at three levels based on the “county–town–village” hierarchy.

## 1. Introduction

Equalization of public services is an ideal goal of a country’s political, economic, and social development [1]. In the process of regional development, providing medical treatment, education, health care, public security, and other services for all residents is a guarantee of fundamental human rights, which can not only improve residents’ well-being but also serve as an input to aggregate economic activities and national output [2]. In 2015, the United Nations launched “Transforming our World: The 2030 Agenda for Sustainable Development” and proposed 17 Sustainable Development Goals. Goals 3, “Good Health and Well-being” and Goal 4, “Quality Education” clearly state the necessity to provide essential health services for people of all ages and ensure inclusive and equitable access to education services for all.

However, the unbalanced supply of public services is a problem that all countries need to solve, and this imbalance is especially prominent between urban and rural areas. Even in developed European and American countries, there are significant differences in the resources and quality of public services such as education and health care between poor rural areas and urban areas with higher income [3]. For developing Asia-Pacific and African countries, the unequal provision of public services between urban and rural areas is more serious [4,5]. In recent years, as a developing country with the largest population in the world, China has made remarkable achievements in poverty alleviation and secured a complete victory in its fight against poverty. However, the unbalanced development in China’s rural areas is still severe, clearly reflected in the income gap, infrastructure construction, medical security, and social security. The imbalance of public services between urban and rural areas in China has become the focus of national and even international attention [6].

In 2005, the concept of equalizing public services was first proposed in China’s 11th Five-Year Plan. Then, the 12th Five-Year Plan was offered to improve the basic public service system covering urban and rural residents and promote equitable access to essential public services. Finally, in the 14th Five-Year Plan, it was pointed out that the equalization of basic public service needs to be improved among regions, urban and rural areas, and different groups of people in China. These relevant national policies show that equalizing public services between urban and rural areas has become essential for China to build moderate prosperity in all respects and achieve shared prosperity. On the other hand, with China’s rapid urbanization process, the urbanization mode is gradually turning to a county urbanization basis.

In 2022, the Chinese government issued “Opinions on Promoting Urbanization Construction with the County Seat as an important Carrier” which proposed to conform to the changing trend of population flow in county seats, complement and strengthen the weakness of county seats, and guide the development direction of county seats by classification. It indicates that county seats have become the main field of urbanization development. Furthermore, it is also emphasized to strengthen the supply of public services and improve the livelihood of county seats, which means that the county will become a critical unit to realize the equalization of public services.

In general, countries worldwide are urgently seeking solutions to the unbalanced allocation of urban and rural public service resources. As a developing country with a significant imbalance between urban and rural areas, China is representative of the unbalanced allocation of public service resources. The development of county urbanization is characterized by Chinese national policy guidelines. The county becomes an integral unit to realize the equalization of public services.

In this context, this paper attempts to analyze the problems and shortcomings of urban-rural public service resource allocation in the context of county urbanization, considering the new population flow trend in counties. We selected 81 sample counties in 28 provinces in China as the primary research unit. We used mobile phone signal data to analyze the population flow trend in the counties by determining the changes in the geographical location of users. At the same time, a large-scale questionnaire and field survey was conducted in the sample counties to summarize the usage and satisfaction of rural residents with public service facilities. Finally, combining the above analysis, we summarize the new demands of rural residents for public services and the shortcomings of public service resource allocation at the county scale. We propose targeted planning strategies and policies to equalize urban-rural public services. The remainder of this paper is organized as follows. The second part is a brief review of the literature regarding the equalization of public services. The third part introduces the study area, methodology, and data resources. The fourth part proceeds with the results and analysis. The fifth part proposes planning strategies and policy recommendations. Finally, the sixth part discusses and concludes this paper.

## 2. Literature Review

The equalization of public services has always been an important research topic, especially in rural areas. As early as the 18th century, Adam Smith proposed in “The Wealth of Nations” that the state has to provide public services fairly [7]. Samuelson also pointed out that public goods should be equally consumed by all people, which implied the ideal of equalization of public services [8]. Subsequent related theoretical studies mainly focus on realizing the equalization of public services and generally emphasize promoting the equalization of public services provided by governments at all levels by increasing public finance expenditure [9,10]. After the 1980s, Cullingford et al. pointed out that the unbalanced public service supply in urban and rural areas was different, so it is necessary to study the unstable public service supply in rural areas [11,12,13].

In developed European and American countries, the problem of unbalanced public service supply in rural areas has not been solved. For example, Cloke and Little studied public transport and retail opportunities concerning different social classes in rural areas of Gloucestershire, England, and they found that the social resources possessed by more affluent social groups in rural areas enable them to have more opportunities to enjoy public services than disadvantaged groups [14]. Similar studies have revealed disadvantages marginalized groups faced, such as the elderly in rural areas, in enjoying public services. More serious is that these disadvantages have been taken for granted in many cases [15,16,17]. In the U.S., there is a significant gap in access to health care between rural and urban residents due to economic conditions, quality of physicians, public transportation, and other conditions [18]. In Latin American countries, educational decentralization has also led to a widening gap in educational quality between schools in rural areas and other regions [19]. Developing countries in the Asia-Pacific region have achieved impressive economic growth in recent decades. Significant improvements in indicators such as primary school enrollment, under-five mortality, and life expectancy are signs of overall progress in education and health services in the region. However, the quality of public services provided by most Asia-Pacific countries is still poor, and there is a huge gap between different regions. As a result, the public service supply in rural areas is difficult to benefit the natural target groups, which is especially obvious in Southeast Asian countries such as Vietnam, India, and Thailand [4,20,21].

In terms of influencing factors of rural public service supply, many studies pointed out that population distribution and mobility trends were essential. For example, Moseley and Packman found that in rural areas of Britain, the population size of settlements could better explain the supply of public services, and large-scale settlements usually had better public service resources [22]. According to the report of the Rural Development Commission, the provision of rural public services is a function of national and local forces, which are particularly affected by population size, age distribution, and socioeconomic characteristics [23]. Kesterton et al. suggested that simply increasing the number of public service facilities did not necessarily solve the problem of inequality of public service resources and that farmers’ economic condition was also an important factor influencing their choice of public services [24]. In addition, Arcury et al. found that improving the mobility of rural residents played an essential role in their access to equitable public services [25]. The research of Abate revealed that geographical distance, especially the last mile, had an important impact on the equalization of public services in rural areas [5]. Other studies pointed out that high-quality practitioners were the key to improve the quality of public services such as education and medical care in rural areas and put forward policy suggestions to relevant departments regarding personnel training and recruitment [3,21]. Central and local government policies also influence rural public services, economic pressures private and public service providers face, etc. [23].

**Figure 1:**
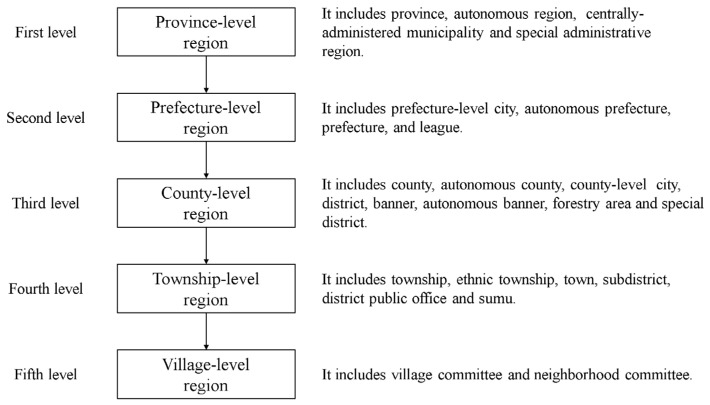
Chinese administrative territorial hierarchy (Source: by the authors).

However, compared with other countries, the equalization of urban and rural public services in China has its particularity caused by China’s unique administrative system (Figure 1). In China’s administrative system, counties have been the basic level of national administration since ancient times. They are precise administrative subjects and the most direct planning platform for implementing policies. In ancient China, there has always been a saying that “the counties are governed, and the world is safe” With the transformation of China’s urbanization process to county urbanization and the new relevant national policies mentioned above, the county will become a vital unit to realize the equalization of public services. In fact, the county is a precise administrative subject with management functions and can take targeted actions to solve the unbalanced allocation of public service resources, and it also includes county seats, townships, and rural areas, which concentrate on the most complex urban and rural issues, and the research results are typical. Therefore, more and more studies on equalizing urban and rural public services in China have begun to pay more attention to the county.

However, obtaining data at the county level is difficult, so most studies still take provinces or cities as the primary research units [6,26,27,28]. Few studies have measured the quality of public services at the county level. Based on county-level data, Deng and Lu analyzed the public service supply efficiency of 38 counties in Chongqing from 2008 to 2011 and 2006 counties in China from 2009 to 2015 [29,30]. Concerning the equalization of public services, some studies explored the influencing factors of public service resource allocation or evaluated the effect of policies on promoting the equalization of public services by regression models or qualitative analysis [31,32,33,34]. Some studies focused on case studies and put forward relevant policy suggestions through in-depth analysis of individual cases [35,36,37]. Others used spatial analysis to evaluate the equalization and efficiency of public service resource allocation and optimize their layout [38,39,40,41].

In general, the research on the equalization of urban and rural public services based on counties reflects the special national conditions of China, which is of great significance in solving the problem of unbalanced development between urban and rural areas. At the same time, it can also provide experience for the international discussion on equalizing public services. Based on the literature review, it is found that existing studies mainly have the following limitations: (1) In selecting a research scale, most of them take cities or provinces as the primary research unit. Few take counties as the object, which shows a lack of pertinence in the context of China’s county urbanization. Moreover, most existing county-level studies focus on the specific type of county or counties within a particular region. Therefore, few studies have covered counties in the whole country. (2) The population flow trend can reflect residents’ actual demand for public services, which is a crucial factor affecting the equalization of public services. However, few studies consider the situation of population flow.

## 3. Study Area and Data Resource

As mentioned earlier, counties are the basic level of the national administration in China and are the most direct coordination platform to achieve equalization of public services. Therefore, this paper selects counties as the research object to improve the inadequacy of existing studies that mainly take the provincial scale as the research unit. At the same time, considering the vast territory of China and the significant differences in natural endowments, economic conditions, and locational environments among counties across the country, a nationwide selection of research subjects is needed to reflect the problems of public service resource allocation and make the research results more representative more comprehensively. However, with more than 1300 counties in China, it is challenging to conduct an in-depth research study on all of them, so this paper considers selecting 2 or 3 sample counties in each province as research subjects and using them as representatives to reflect the situation in that province. Furthermore, to ensure that the sample counties selected in each province can represent the general level of the rural areas, the selected counties are mainly located in the central agricultural production areas, and the economic development level is at the average level of the province.

**Figure 2:**
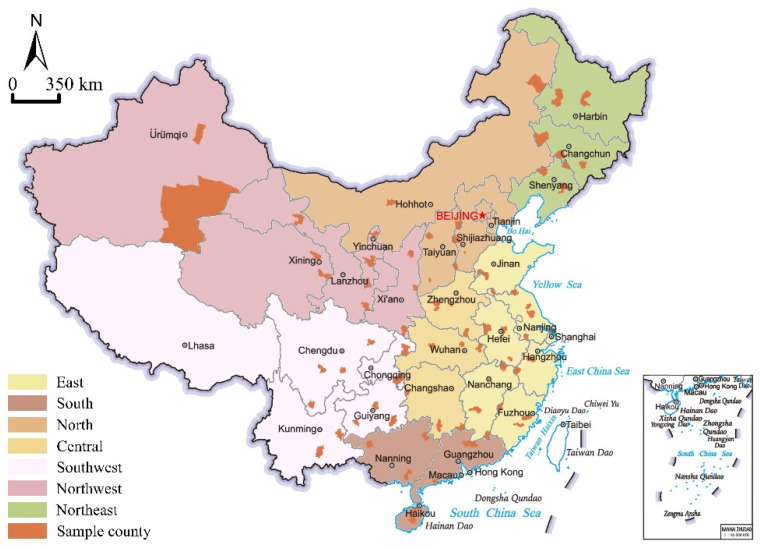
Overview of the study area (Source: by the authors).

Moreover, we avoid selecting counties with a high level of economic development dominated by industries such as manufacturing or tourism. Based on this, 81 sample counties in 28 provinces were selected in this study, of which 2–3 counties were selected in each province (Figure 2). Table 1 shows the information and basic situation of sample counties in the seven regions. Overall, the sample counties selected for this study can reflect the level of development of each region. As the most economically developed region of China, East China also has the highest level of rural development, with a large county population and significantly higher per capita GDP and disposable income per rural resident than other regions. Central and North China are dominated by plain terrain, the counties there also have large populations, and the economic development level is in the middle of the whole country. Northwest China is sparsely populated, and the counties there have a small resident population and a low level of economic development. The average resident population and average GDP per capital of its sample counties are both lower than other regions.

In addition, according to the characteristics of geography, climate, humanities, and economy, the country’s land area is divided into seven regions in complete reference to China’s geographical divisions: East China, South China, North China, Central China, Southwest China, Northwest China, and Northeast China. We summarize and compare the characteristics of urban-rural population flow trends and the shortcomings of public service resource allocation in different regions and make targeted recommendations. In short, this study is conducted on behalf of 81 sample counties in 28 provinces, which achieve full coverage of all regions nationwide and can reflect the overall situation of China’s county public service resource allocation and the status of each area comprehensively and objectively.

The research team conducted the National Rural Construction Evaluation in 2021 for these 81 sample counties and collected a large amount of data, including local statistics, questionnaire data from villagers and village cadres, and mobile phone signal data. The local statistics include data on the economic development level, farmhouse construction, village construction, and county construction of the 81 sample counties in 2020, obtained from each county’s relevant departments.

The research team conducted a questionnaire survey among villagers and village cadres in the 81 sample counties to investigate the allocation of rural public service facilities and the usage and satisfaction of rural residents with these facilities. The respondents assigned to the villagers’ questionnaire are the residents who have lived in the village for a long time. Moreover, the respondents of the village cadres’ questionnaire are the members of the village committees, who are familiar with village affairs. Each administrative village is required to finish only one village cadre questionnaire. To facilitate the collection of questionnaires, the research team built a mobile working platform for collecting villagers’ and village cadres’ questionnaires in the form of online questionnaires. Respondents completed the questionnaire by logging on to this mobile working platform. All the questionnaires were collected under the organization of the county government of the 81 sample counties. With the organization of the county government, the township government department organized a specified number of villagers and village cadres to complete the questionnaire. Each township in the sample counties needs at least 80 villagers to finish the questionnaire. In addition, three townships in the sample counties need to organize village cadres from all administrative villages to finish the village cadres’ questionnaire. The survey content of the villagers’ questionnaire includes respondents’ satisfaction with the use of public service facilities and service quality at the county, town, and village levels and includes the allocation of public service resources in administrative villages. Their complete structures and main contents are shown in Table 2. To ensure the reliability of the collected questionnaires, the research team cleaned all the questionnaire data and finally collected a total of 146,511 valid villagers’ questionnaires and 3993 valid village cadres’ questionnaires.

**Figure 3:**
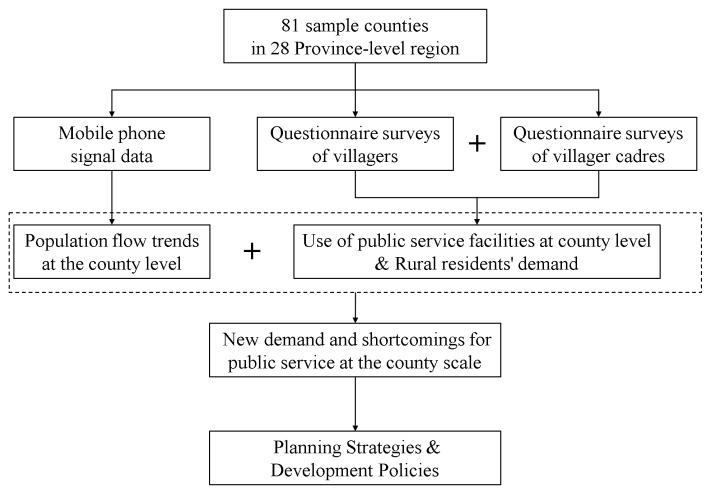
The analytical framework and methodological procedures of this study (Source: by the authors).

Mobile phone signal data provided by China United Network Communications Group Co., Ltd. contains subscriber data for all townships in 81 sample counties between January 2020 and July 2021, which can be used to determine population flow based on changes in the geographical location of subscribers. In addition, we also traveled to Guangdong, Hunan, Hubei, Fujian, Shanxi, Hainan, and other provinces to conduct discussions with local county education bureaus, health care bureaus, and other relevant government departments, conduct field surveys in townships and villages, and interview township cadres, village cadres and villagers to understand the allocation of public service resources, service quality, as well as rural residents’ satisfaction with public services and their suggestions. Figure 3 shows the analysis framework and the methodological procedures of this study.

## 4. Results and Analysis

### 4.1. Concentration and Dispersion of County Population

In this paper, we analyzed the mobile phone signal data of 81 sample counties in China between January 2020 and July 2021 to summarize the population flow trend in China’s counties.

#### 4.1.1. Population Outflow from the County

The mobile phone signal data show that during 2020–2021, the number of permanent populations decreased in all 81 sample counties, with the average number of outflow population in the county reaching 47,792 and the average outflow population as a percentage of the permanent population reaching 13.29%. The outflow from counties in Xinjiang (22.34%), Hubei (18.37%), Guizhou (18.29%), Chongqing (16.52%), Henan (15.89%), Yunnan (15.48%), and Fujian (15.24%) province is evident, and the proportion of outflow from counties to the permanent population is higher than 15%.

In terms of age structure, the county outflow population is dominated by the young and middle-aged population aged 19–39. Among the 81 sample counties, the average percentage of the outflow population aged 19–29 reached 44.63%, and the average rate of those aged 30–39 reached 17.97%. Yunnan (72.64%), Guizhou (72.50%), and Guangdong (70.76%) provinces have the most pronounced outflow of young and middle-aged people.

#### 4.1.2. Increased Population Agglomeration Capacity of the County Seat

**Figure 4:**
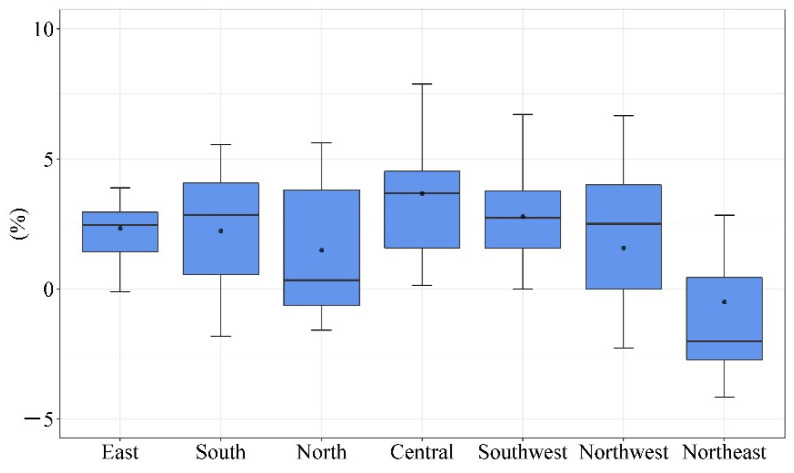
The value of the change in population agglomeration degree of the county seat in the seven regions during 2020–2021 (Source: by the authors).

The indicator “county population agglomeration degree” (county seat permanent population/county permanent population) is used to reflect the population agglomeration ability of counties. The mobile phone signal data show that the population agglomeration capacity of county seats in 81 sample counties generally increases between 2020 and 2021, and the average value of county population agglomeration degree increases from 45.97% to 48%, with 60 counties having an increase in this indicator. Figure 4 illustrates the change in population agglomeration degree in sample counties within the seven regions. The population agglomeration capacity of county seats in Central China and Southwest China has increased. The average county population agglomeration degree has risen by more than 3%; however, the population agglomeration capacity of county seats in Northeast China tended to weaken.

The mobile phone signal data show that among the 81 counties in 2020, an average of 11.55% of the outflow population returned to the county in 2021. An average of 41.25% of the returning population returns to the county seat for employment and living. It indicates that the county seat is becoming the main concentration of the returning population. Regarding age structure, the average percentage of the returning population aged 19–29 is 34.29%, which is lower than the average percentage of the same age group in the outflow population (44.63%). On the other hand, the average rate of the returning population aged 30–39 is 25.61%, which is higher than the average percentage of the same age group in the outflow population (17.97%). This indicates that at this stage, the returning population in the county is dominated by middle-aged and young people aged 19–39.

#### 4.1.3. Hollowing Out and Aging of Rural Areas

The questionnaire survey of village cadres shows that an average of 14.6% of administrative villages in 81 sample counties show a high degree of hollowing out (the ratio of permanent population to household registration population is less than 30%). The average proportion of the resident population to household registration population in rural areas of the county is 0.65, with evident population outflow from rural areas and severe hollowing out of villages.

According to the questionnaire survey of village cadres, the average proportion of the elderly population aged 65 or older in the permanent rural population of 81 sample counties is 25.8%, which is near twice the proportion of the people in the same age group (13.5%) in the seventh national population census of China. The mobile phone signal data show that the average proportion of the permanent population aged 60 years or older within the township and village is 14.2%. In contrast, the average ratio of the permanent population in this age group within the county seat is only 9.5%. This indicates that the aging of the people in rural areas of China is very prominent.

### 4.2. County Public Services under the Recent Trend of Population Flow

As mentioned above, at this stage, the population flow in China’s counties shows a new trend of “overall dispersion and partial concentration”, which means that under the general direction of continuous population outflow from counties, especially from rural areas, the population within counties and the returning population have started to concentrate in the county seats.

#### 4.2.1. High Expectations of the County’s Education and Medical Services

**Figure 5:**
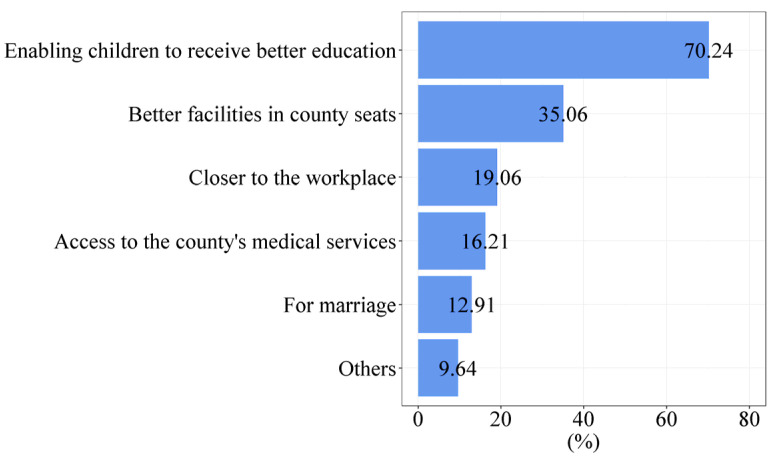
Reasons rural residents buy commercial houses in the county seat (Source: by the authors).

The questionnaire survey of villagers shows that the proportion of rural students attending elementary, middle, and high school in county seats in the 81 sample counties is 26.75%, 35.21%, and 59.38%, respectively. This means it has become common for rural students to go to the county to receive education, especially at the high school level. According to the statistics, 53.79% of elementary and middle school students in 81 sample counties are from rural areas. Now, enabling children to receive higher educational services has gradually become the most crucial reason rural families move to the county seat. According to the questionnaire of villagers, Figure 5 illustrates the motivation of the interviewed villagers in the sample counties to purchase a commercial house in the county. Of those 28,665 rural residents who have already purchased a house in the county seat, 70.24% said that their reasons for buying a place in the county seat include “enabling children to receive a better education”.

**Figure 6:**
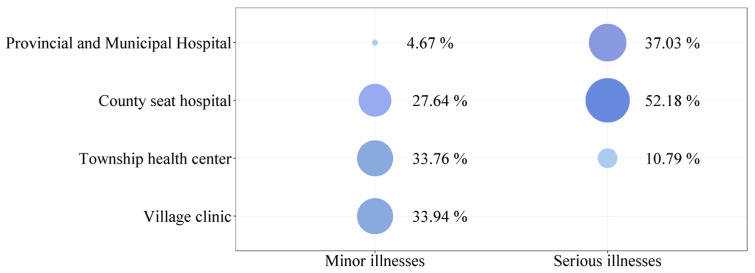
Rural residents’ choice of location for medical and health care (Source: by the authors).

In addition to educational services, better medical services are also essential in attracting rural residents to move to the county. According to the questionnaire of villagers, 16.21% of those rural residents who had already purchased a house in the county seat said that their reasons for buying a house in the county seat included “access to the county seat’s medical services” Figure 6 shows the choice of the location of medical treatment for different diseases among the interviewed villagers in the sample counties. Among rural residents, 52.90% said they would go to the county seat for medical care when seriously ill, indicating that medical services in the county seat play a vital role in the whole county.

Therefore, with the increasing demand of rural residents for education and medical service levels of county seats, increasing the allocation of education and medical service resources and improving their quality is essential to promote the construction of urbanization with the county seat as the carrier. However, the level of education and medical services in the county seat has not yet met the needs of rural residents, and there are still apparent shortcomings.

**Figure 7:**
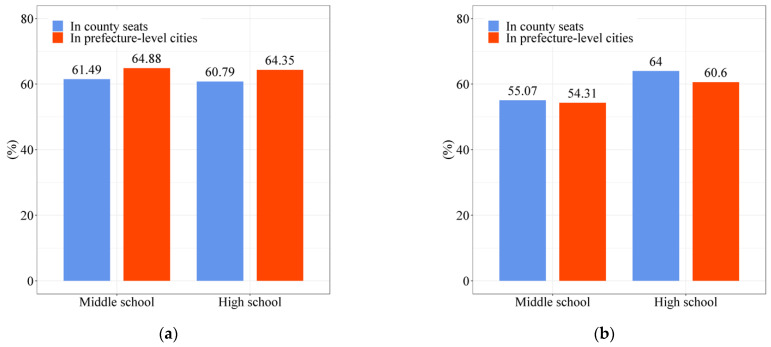
Rural residents’ satisfaction with the schools their children attend. (**a**) Rural residents’ satisfaction with the quality of teaching in their children’s schools; (**b**) rural residents’ satisfaction with the accommodation of the schools their children attend (Source: by the authors).

In terms of education services, according to statistics, the percentage of candidates admitted to Project 985 and Project 211 universities in the 81 sample counties in 2020 is only 3.84%, which is lower than the average level of the prefecture-level cities (7.43%) to which these counties belong. According to the questionnaire of villagers, Figure 7 shows the satisfaction rate of rural residents with the quality of education and boarding conditions of schools in county seats and prefecture-level cities (the proportion of respondents who chose “satisfied” and “relatively satisfied” to the total number of people). There are 26,049 children of the villagers interviewed enrolled in high schools in county seats. Among the respondents, 61.49% and 55.07% of them are “satisfied” or “relatively satisfied” with the quality of education and boarding conditions of middle schools in the county seat, and 60.79% and 54.31% of respondents are “satisfied” or “relatively satisfied” with the quality of education and boarding conditions of high schools in the county seat, both of which are lower than the satisfaction for schools in the prefecture-level city. In general, education resources in county seats are relatively tight, and there is still a certain threshold for rural students to attend high school in the county seat. In addition, there is a large gap between the quality of education services in the county seat and the city.

In terms of medical services, according to statistics, the average number of beds in medical institutions per 1000 people in 81 sample counties is 5.5 per 1000 people. The average number of licensed physicians per 1000 people is 2.4 per 1000 people, both of which are lower than the intermediate level of the municipal districts of the prefecture-level cities (9.0 per 1000 people and 4.0 per 1000 people) to which these counties belong. The questionnaire survey of villagers shows that 36.4% of rural residents would choose to go to provincial and municipal hospitals for better medical care when suffering from a severe illness. As mentioned above, the county seat has gathered 48% of the counties’ population and is the first choice of more than half of rural residents with severe illnesses. The population served by hospitals in county seats is much larger than the permanent population, the mismatch between the supply and demand of medical resources in the county seat is apparent, and the quality of medical services needs to be improved.

#### 4.2.2. The Township Assumes a Crucial Public Service Function

**Figure 8:**
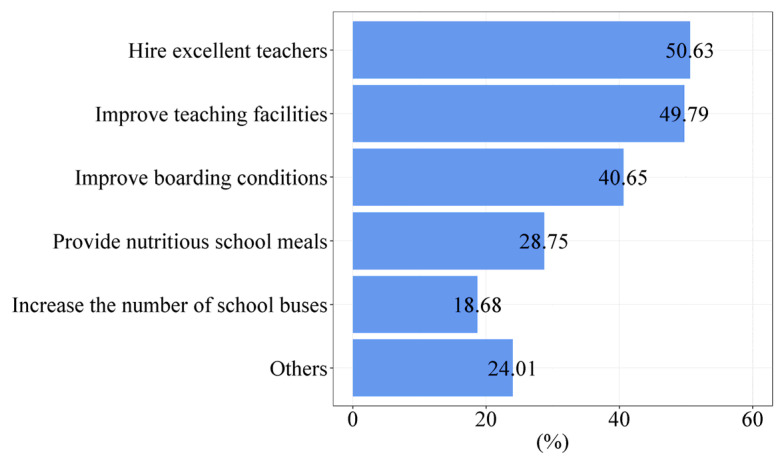
Areas where rural residents think the township elementary school their children attend needs improvement (Source: by the authors).

With the continuous population exodus from rural areas, more and more rural children will study with their parents in the county seat or other locations outside the county. Many rural elementary schools are being abolished due to insufficient students to sustain their operations. The questionnaire survey of villagers shows that the proportion of rural children attending elementary school in townships is 51.19% in 81 sample counties. It has become a significant trend for rural children attending townships’ elementary schools. However, at this stage, the teaching facilities’ support and education service quality of China’s rural elementary schools still have apparent shortcomings. Figure 8 illustrates where rural residents think the township elementary school their children attend needs improvement. A total of 47,344 villagers interviewed have children attending elementary school in their township or neighboring townships, of which 50.63%, 49.79%, 40.65%, and 28.75% of them think the quality of teachers, teaching facilities, school meals, and boarding conditions need improvement.

In terms of medical services, according to the questionnaire survey of villagers, an average of 33.64% of rural residents in the 81 sample counties choose to visit township health centers when they suffer from minor illnesses. This reflects that township hospitals also undertake part of the daily medical services for rural residents. In addition, according to the current epidemic prevention and control policies, village clinics are generally not allowed to receive patients with fever. Township hospitals must receive these patients and carry out the large-scale nucleic acid testing in rural areas. Therefore, the township hospital plays a vital role in preventing and controlling the epidemic in rural areas of China.

#### 4.2.3. Needs of the Elderly and Children in Villages Must Be Safeguarded

In rural areas, with the continued exodus of young and middle-aged laborers, children and the elderly are the main groups left behind to live in villages long-term. Therefore, their needs for education, elderly care, and medical services need attention. The questionnaire survey of villagers shows that the average percent of rural children studying in kindergartens and elementary schools of administrative villages is 32.19% and 26.85%, respectively. This means that there are still many rural children’s needs for education services in villages that must be guaranteed. However, it has become a significant trend for rural children to move out with their parents or go to townships to study.

**Figure 9:**
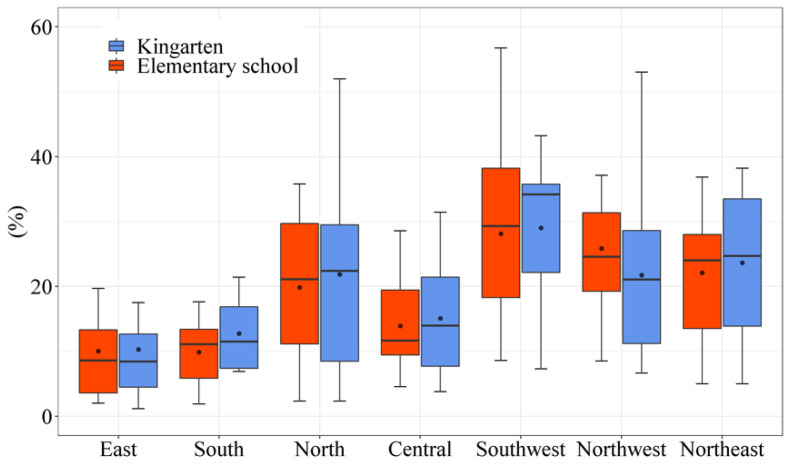
The percentage of administrative villages without elementary schools or kindergartens within more than 30 min travel time in the seven regions (Source: by the authors).

At present, the most apparent contradiction of village-level education services is the necessity of guaranteeing the needs of rural children for education services and the difficulty of maintaining the operation of rural schools with a small number of students. It is not practical to set up a kindergarten or elementary school for only a few students in an administrative village, which leads to the closure of a few village kindergartens and elementary, and students in remote villages can only choose to go to the nearby village or the township to receive an education. According to the statistics, only 35.69% of administrative villages in the 81 sample counties had universal kindergarten on average in 2020. The questionnaire survey of village cadres shows that, on average, 19.31% and 19.15% of administrative villages in the 81 sample counties are more than 30 min away from the nearest kindergarten and elementary school, which means that a few rural students still face the problem of distance to school. Based on the data from the village cadres’ questionnaire, Figure 9 shows the percentage of administrative villages without elementary schools or kindergarten within more than 30 min of travel time in the sample counties of the seven regions. We can find that the percentage of administrative villages without elementary schools or kindergartens within more than 30 min of travel time in Southwest, Northwest, Northeast, and North China is significantly higher than in the other three regions. This is because in these four regions, the distance between villages and kindergartens and between villages and elementary schools is longer, and it is more difficult for rural students to go to school than in the other three areas.

The aging problem in rural areas of China is serious. With the continuous outflow of the young and middle-aged labor force, the elderly left behind in rural areas are not cared for, and the pension problem is becoming increasingly prominent. At present, the supply of elderly services in rural areas is insufficient [42]. Statistics show that in 2020, the proportion of administrative villages equipped with elderly service facilities in 81 sample counties was 43.86% on average. In terms of the use condition and service quality of elderly service facilities, the questionnaire survey shows that 30.62% of villagers think the utilization rate of elderly service facilities in their villages is high. On the other hand, only 9.84% of the villagers said they would receive elderly care services in the village, and 53.84% were inclined to choose home-based care. This shows the problem of insufficient resource allocation and low quality of village elderly service in China.

Yangxi County belongs to Yangjiang City and is located in the southwest coastal area of Guangdong Province. It covered an area of 1435 square kilometers and had a permanent resident population of 434,000 in 2020, with an urbanization rate of 43%. In 2020, the GDP of Yangxi County was 28.182 billion yuan, with the fishery industry as the primary industry. The economic development level of Yangxi County is above the middle level among counties outside the Pearl River Delta in Guangdong Province.

**Figure 10:**
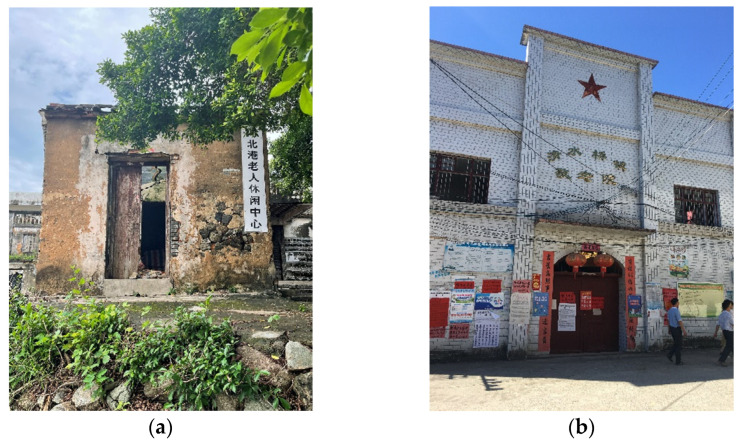
Abandoned village elderly activity center. (**a**) Scene of a village in Yangxi County, Guangdong Province; (**b**) scene of a village in Ningyuan County, Hunan Province (Source: by the authors).

Currently, the aging of the rural population in Yangxi County is serious. The questionnaire of village cadres shows that the elderly population over 65 years old in rural areas accounts for 22.7%. In order to ensure the demand of the rural left-behind elderly for old-age care services, the county authorities required the construction of at least one elderly service facility in every administrative village. However, our field research found that the use of village elderly service facilities in Yangxi County is not good. Although all 131 administrative villages have elderly service facilities, only eight of them can provide elderly care services. In contrast, the other facilities provide lunch for the elderly or serve as a place for daily entertainment. Due to a lack of operating funds and staff, some village facilities are no longer providing services or have been closed for a long time. The same phenomenon has been found in Hunan, Qinghai, and other provinces. Figure 10 shows two abandoned village elderly activity center found during our field research.

In addition, village-level medical services are vital for the health of left-behind children and the elderly. A total of 34.05% of the villagers said they would prefer to go to the village clinic when suffering from minor illnesses. At present, almost all administrative villages in China have village clinics. Statistics show that an average of 95.37% of administrative villages in 81 sample counties have at least one village clinic, and 88.06% of villagers said that the village clinic in the village they live in always has a doctor available (except on weekends and holidays) and that the villagers’ needs for medical treatment can be met at the village clinic. What is more, the villagers’ average satisfaction with the level of medical services at the village clinics has reached 56.15%. However, according to statistics released by the National Health Commission, there were 60,828 village clinics in China in 2020, a decrease of 7266 from 2019 and a total decrease of about 30,000 in the last five years.

The reason for this is that, on the one hand, with the accelerated urbanization process, some village clinics have been upgraded to community health service stations. On the other hand, there is a significant departure of the rural population, a decrease in the number of patients, serious aging of the pastoral doctor team, and a lack of new members to replenish them, making it difficult for village clinics to maintain operations. Through a field survey, we learned that village doctors in China generally face a poor working environment and low salary, resulting in a low willingness of young doctors to work as village doctors [35].

**Figure 11:**
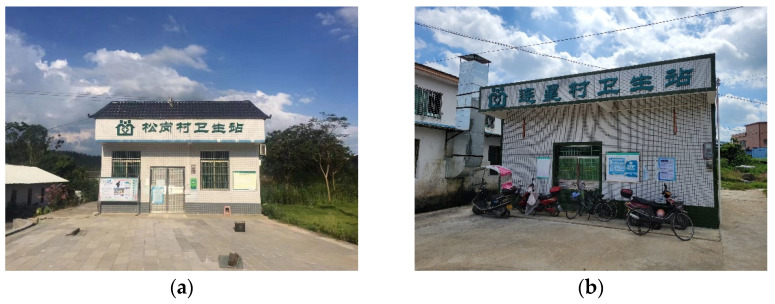
Village health office without village doctors on duty. (**a**) Scene of a village in Wengyuan County, Guangdong Province; (**b**) scene of a village in Lianping County, Guangdong Province (Source: by the authors).

Wengyuan County belongs to Shaoguan City, located in the northern mountainous area of Guangdong Province, with an area of 2175 km^2^. In 2020, the permanent resident population was 322,000, with an urbanization rate of 35%. Presently, Wengyuan County has realized the full coverage of administrative village clinics. However, according to the county health department staff, the number of administrative village doctors in remote areas of the county is insufficient. As a result, some villages still face the situation of no village doctors. Currently, the county health department has tried to send doctors from neighboring community health service stations to these remote villages at regular intervals, but the results are not good. During the field survey, we also observed that some village clinics were closed on weekdays, and there was no village doctor on duty. Furthermore, the villagers’ questionnaire survey results showed that only 18.49% of the villagers in Wengyuan County would choose to visit the village clinic when they had a minor illness, and the villagers’ satisfaction with the service quality of the village clinic is not high. In general, the allocation of rural medical service resources in China has been relatively complete, but there is a greater risk of a shortage of village doctors in the future. Figure 11 shows two village health office without village doctors on duty during our field research.

## 5. Planning Strategies and Development Policies

With the growing demand of rural residents for a better life, relying solely on village-based equalization can no longer meet their needs for public services. The above analysis shows that rural residents have different requirements for public service supply in counties, townships, and villages. Therefore, this study proposes targeted planning strategies and policy recommendations for allocating public service resources in counties based on the “county–township–village” hierarchy.

### 5.1. County Level: Focus on Improving the Quality of Education and Medical Services

County seats are the carriers of county urbanization. The above analysis also reflects that education and medical services in county seats are essential factors in attracting rural residents and returning populations to cluster in county seats. In terms of planning strategies, it is recommended that the county seat’s status as a public service center for the whole county be highlighted in the county’s integrated planning. The improvement of the county seat’s education and medical service capacity should be included as a development guideline to guide the construction of the county seat’s public service facilities. The demand of rural residents should be considered when determining the scale of public service facilities in the county seat, and service provision capacity should be improved. It is recommended that the county’s health bureau and education bureau appropriately increase the standards for the number of healthcare facility beds per 1000 people and the number of occupational physicians per 1000 people for county hospitals, and the standards for the student–teacher ratio of county schools. The county’s educational and medical service resources can benefit more of the county’s population. Moreover, the county’s natural resources bureau needs to ensure the area of construction land for schools and hospitals in county seats when preparing the county’s territorial spatial plan. While meeting current needs, a particular land scale should be set aside to meet the continued growth in demand for education and medical services.

In terms of policy recommendations, we suggest that the county’s health bureau and education bureau adopt policies to improve the quality of education services and medical services in the county seat and narrow the gap in service quality between the county and the city. It is effective to actively carry out distance education and telemedicine services by forming partnerships with schools and hospitals at the municipal and provincial levels. Through these means, the county population can enjoy the city’s education and medical service resources. The trend of population clustering in county seats in Central and Southwest China is more prominent, and the increase of county population agglomeration degree in the sample counties is more than 3% during 2020–2021, which is significantly higher than the other five regions. Therefore, Central China and Southwest China counties need to improve education and medical services in their county seats.

### 5.2. Township Level: Strengthening Boarding Elementary Schools in the Township

At present, townships assume a crucial educational service function in rural areas of China. However, there is still a significant gap between the service level of township elementary schools and the needs of rural students. So, there is a need to strengthen boarding elementary schools in the township. In terms of planning strategies, this paper argues that we should ensure that each township has at least one boarding elementary school and that the vast central townships (>30,000 people) can be equipped with two to three elementary schools according to the actual situation. According to the current standard for the planning of the rural area, public facilities in China, and the actual situation of each region, the counties’ natural resources bureaus need to ensure that the land area target for township elementary schools should not be less than 1.2 m^2^/person. It is also recommended that the service radius of the township boarding elementary school should be reasonably expanded according to the spatial distribution of villages within each township and the schooling needs of rural children. For administrative villages far from the township with a high demand for schooling, accessibility to the township elementary school can be improved by providing school buses. The county’s transportation bureau should guarantee the supply of school buses and reasonably plan the routes of school buses. The county’s education bureau should determine the specific operating arrangements of school buses.

In terms of policy recommendations, we suggest that the county’s education bureau expand the talent pool of township teachers and enhance the quality of teaching in township elementary schools by raising the level of remuneration of township teachers and increasing the number of temporary teachers. On the other hand, the county education bureaus should improve the hardware facilities in township elementary schools, narrow the gap between them and county seats’ schools, and achieve equalization of education services between urban and rural areas. The focus is on improving teaching facilities and boarding conditions, including facilities for living necessities such as toilets and showers, the sanitary environment of canteens, and the quality of school meals. At present, in some areas such as Chengmai County in Hainan Province, distance education in township schools has begun to be gradually implemented, and distance learning equipment has been deployed in central township schools to improve the quality of teaching in township schools, which is also a method worthy of reference for other areas.

### 5.3. Village Level: Meeting the Needs of the Elderly and Children in Villages

#### 5.3.1. Guarantee Rural Early Childhood Education Services

With the population exodus from rural areas, the problem of rural education facilities struggling to operate due to a lack of students is particularly acute. Children in the preschool stage are still young. They lack the necessary self-care skills, making it inconvenient for them to travel to surrounding villages to attend kindergarten. However, due to operational costs, most regions have certain restrictions on the size of administrative villages equipped with kindergartens. For example, Guangdong Province requires administrative villages with a resident population of more than 4000 to set up kindergartens, and Jiangxi Province requires more than 2000. According to the policy requirements, the vast majority of administrative villages are not necessary to set up kindergartens. Therefore, it is recommended that counties take diversified measures to guarantee early childhood education services in the village according to the actual local situation.

In terms of planning strategies, this paper argues that it is not necessary to compel each administrative village to be equipped with a kindergarten. Instead, according to the current standard for the planning of rural area public facilities in China and the actual situation of each region, kindergartens should be required in large (1001–3000 people) or extensive (>3000 people) administrative villages. It also ensures that the proportion of construction land for the village’s kindergartens should be kept between 0.4% and 1.1%. At the same time, when we determine the service capacity of the kindergartens in central villages, we also need to consider the needs of the children in adjacent villages. If necessary, the kindergartens also need to be equipped with school buses. For administrative villages that do not have the conditions to set up kindergartens, one suggestion is to use the teaching facilities and teachers of village elementary schools to open kindergartens attached to them through new construction or renovation. Suppose there is already a preschool attached to the village elementary school. In that case, we can refer to the experience and practice of Yunnan Province and take the measure of “changing the class into a kindergarten” to turn the preschool into an independent kindergarten. Second, we encourage the establishment of private kindergartens with the help of social forces. By establishing a clear review and assessment mechanism, the local government can ensure that private kindergartens operate in compliance. Meanwhile, local governments subsidize funds for standardized private kindergartens that pass the examination to reduce operational pressure. In Southwestern, Northwestern, Northeastern, and Northern China, the understaffing of village kindergartens is even more pronounced, so extra attention needs to be paid to securing rural early childhood education services in these regions.

#### 5.3.2. Promote the Sinking of Medical Resources to Rural Areas

Many studies have mentioned that better access to medical services must become priorities in rural area, and service providers becoming key figures in better access to care must become priorities in deprived areas [21,25,43]. Almost all administrative villages in China have village clinics, but the major problem is the shortage of rural medical personnel. Firstly, local governments can attract young doctors to join the rural medical staff by improving village doctors’ treatment and welfare subsidies and allocating all the medical equipment in village clinics. The second is to promote the construction of a county medical community, organize county hospitals and township health centers to provide targeted support to villages, and regularly go to villages to provide rural residents with home treatment services.

#### 5.3.3. Provide Village Elderly Services According to Actual Needs

The problem of hollowing out and aging in China’s rural areas is prominent, and the elderly left behind in rural areas need to be addressed urgently. However, due to the influence of traditional Chinese culture, aging in place is still the most mainstream way of aging in Chinese rural society. In fact, except for a minimal number of older people who are seriously ill, the majority of older people left behind are capable of taking care of themselves. So, providing them with nutritious and healthy meals and places for daily recreation can already meet most of the needs of the elderly. Therefore, it is recommended that the civil affairs department gradually increase the operating subsidies for rural elderly service facilities to guarantee the price and quality of daily meals. At the same time, tables, chairs, TVs, radios, and other facilities should be configured to enrich the daily lives of the elderly left behind. In addition, local governments can join the village committee to use the unused farmhouses in the village to provide senior care services to reduce construction costs. For the elderly left behind with serious illnesses or mobility problems, local governments can establish personal files, and organize caregivers to visit their homes regularly and provide elderly care services.

## 6. Discussion and Conclusions

Achieving equalization of public services is an ideal goal shared by all countries worldwide. However, the problem of uneven supply of public services has not yet been solved, especially in rural areas in developed European and American countries and other developing countries. A large number of studies based on theoretical analysis or empirical research focus on measuring the quality of public services and achieving the equalization of public services. Unlike other countries, China’s administrative system is based on counties at the primary level, making it unique in equalizing public services. In the current process of county urbanization that China is in, the county will become a fundamental unit in realizing equalizing public services. Therefore, based on relevant studies, this paper takes counties as the primary research unit to study the new trends of population mobility in China’s counties. We combine the large-scale questionnaire surveys and field research, analyze the new demands of rural residents for public services, and summarize the shortcomings of public service resource allocation at the county scale. The marginal contributions of this paper are divided into two main aspects. First, the study is conducted with counties as the basic unit, which fully reflects China’s unique national conditions and provides an empirical reference for the international discussion on public service equalization. The second is to analyze and summarize the current shortcomings of allocating urban and rural public service resources in counties based on the residents’ needs, considering the trend of population flow in counties and a large-scale questionnaire survey. This will make the proposed planning strategies and policy recommendations more relevant and informative.

This paper agrees with existing studies that population distribution and mobility trends are important factors affecting public service supply. Therefore, it is of positive significance for us to grasp the population flow trend to equalize public service resources in urban and rural areas [21]. Understanding population flow trends enables decision makers to know the priority areas that need to be developed in the future and allows for more targeted planning strategies. In this study, through the analysis of mobile phone signal data and questionnaire data, we found a clear tendency for the county population to cluster in the county seat. At the same time, many rural children choose to attend township schools. This supports the decision to focus on improving public services in county seats and strengthening the construction of township schools in the future. In addition, by conducting in-depth questionnaires for individual villagers, we can understand rural residents’ use of public service facilities and their satisfaction. We can also identify the shortcomings of the current allocation of public service resources in urban and rural areas more accurately.

The study found that the problem of inequitable access to public services for marginalized rural groups, which exists in other countries, is equally severe in China [15,16,17,20]. As the rural exodus of young adults continues, the needs of the two vulnerable groups, left-behind children and the elderly, are not being taken seriously, and early childhood education services and elderly services at the village level are in urgent need of improvement. In addition, in rural areas of China, facilities for medical services and compulsory education services are relatively good, and rural residents mostly have easy access to these services. However, the main problem is a significant gap in the quality of the services they receive compared to urban areas. A typical example is the high coverage of village clinics but the shortage of village doctors. Therefore, this paper holds the same view as Pagaiya and other scholars that improving the level of relevant practitioners is the key to improving the quality of medical and compulsory education services in rural areas [3,18,21].

Compared with the studies on measures to equalize public services, this paper proposes targeted planning strategies and policy recommendations for allocating public service resources at the county, township, and village levels, based on China’s unique “county–town–village” hierarchy [35,36,37]. In addition, this paper recommends improving the quality of education and health care services in the county and strengthening the construction of boarding schools in the townships, which has rarely been mentioned in other studies. As for the allocation of village public service facilities, this paper is consistent with existing studies, which argue that it is unnecessary to mandate that each administrative village be equipped with a kindergarten or elderly service facility. Instead, this paper argues that the layout should be reasonable according to the population distribution and that the existing resources in the village should be integrated to provide services in a diversified way.

However, there are still some limitations to this study. China is a vast country, and different counties’ geographical locations and development stages vary greatly. Mountainous counties with inconvenient transportation and more developed counties near large cities may also impact the strategy of their public service resource allocation. China’s national policy also mentions the need to classify counties and equalize public services by category. However, due to the limitation of article length, different types of counties are not analyzed and discussed in this paper. In the future study, we will select different types of sample counties for comparative analysis and propose more targeted planning strategies.

## Figures and Tables

**Table 1:** Information and basic situation of sample counties in the seven regions.

Region	The Number of Sample County	Basic Situation		
		Average Resident Population (Person)	Average GDP per Capital (10,000 Yuan/Person)	Average Disposable Income per Rural Resident (Yuan)
East	18	545,718	6.7	20,500
South	8	354,627	3.8	15,825
North	8	244,121	4.3	15,293
Central	9	451,107	4.1	14,409
Southwest	12	455,373	4.4	15,324
Northwest	16	191,572	3.6	15,497
Northeast	10	271,799	4.2	17,165

Note: The relevant data are summarized from the counties’ statistics of year 2020.

**Table 2:** The structure and main contents of villagers’ questionnaires and village cadres’ questionnaires.

Villager’s Questionnaire		Village Cadre’s Questionnaire	
Structure	Main Contents	Structure	Main Contents
Basic family information	Number of family members, employment, income, etc.	Basic information	Basic information of the village, including land use, income, etc.
Agricultural production	Household farming situation.	Population	Demographic information of the village, including total number, age structure, etc.
Public service	The use of and satisfaction with public service facilities in the county, etc.	Housing	Housing information of the village, including total number, security, etc.
Housing	Construction of farm buildings and supporting facilities.	Public service	The allocation of public service resources in villages.
Habitat	Sewage treatment and garbage treatment in households.	Habitat	Habitat of the village, including sewage treatment, garbage treatment, etc.
Village Governance	Villagers’ participation in village governance.		

## Data Availability

Not applicable.
